# Increased Programmed Death-Ligand 1 is an Early Epithelial Cell Response to *Helicobacter pylori* Infection

**DOI:** 10.1371/journal.ppat.1007468

**Published:** 2019-01-31

**Authors:** Loryn Holokai, Jayati Chakrabarti, Taylor Broda, Julie Chang, Jennifer A. Hawkins, Nambirajan Sundaram, Lydia E. Wroblewski, Richard M. Peek, Jiang Wang, Michael Helmrath, James M. Wells, Yana Zavros

**Affiliations:** 1 Department of Molecular Genetics, Biochemistry, and Microbiology, Cincinnati OH, United States of America; 2 Department of Pharmacology and Systems Physiology, University of Cincinnati, Cincinnati OH, United States of America; 3 Division of Developmental Biology, Cincinnati Children’s Hospital Medical Center, Cincinnati OH, United States of America; 4 Center for Stem Cell and Organoid Medicine, Cincinnati Children’s Hospital Medical Center, Cincinnati OH, United States of America; 5 Department of Biomedical Engineering, University of Cincinnati, Cincinnati OH, United States of America; 6 Department of Pediatric Surgery, Cincinnati Children’s Hospital Medical Center, Cincinnati OH, United States of America; 7 Division of Gastroenterology, Department of Medicine, Vanderbilt University Medical Center, Nashville, TN, United States of America; 8 Department of Pathology and Lab Medicine, University of Cincinnati College of Medicine, Cincinnati OH, United States of America; University of Illinois, UNITED STATES

## Abstract

*Helicobacter pylori (H*. *pylori)* is the major risk factor for the development of gastric cancer. Our laboratory has reported that the Sonic Hedgehog (Shh) signaling pathway is an early response to infection that is fundamental to the initiation of *H*. *pylori*-induced gastritis. *H*. *pylori* also induces programmed death ligand 1 (PD-L1) expression on gastric epithelial cells, yet the mechanism is unknown. We hypothesize that *H*. *pylori*-induced PD-L1 expression within the gastric epithelium is mediated by the Shh signaling pathway during infection. To identify the role of Shh signaling as a mediator of *H*. *pylori-*induced PD-L1 expression, human gastric organoids generated from either induced pluripotent stem cells (HGOs) or tissue (huFGOs) were microinjected with bacteria and treated with Hedgehog/Gli inhibitor GANT61. Gastric epithelial monolayers generated from the huFGOs were also infected with *H*. *pylori* and treated with GANT61 to study the role of Hedgehog signaling as a mediator of induced PD-1 expression. A patient-derived organoid/autologous immune cell co-culture system infected with *H*. *pylori* and treated with PD-1 inhibitor (PD-1Inh) was developed to study the protective mechanism of PD-L1 in response to bacterial infection. *H*. *pylori* significantly increased PD-L1 expression in organoid cultures 48 hours post-infection when compared to uninfected controls. The mechanism was cytotoxic associated gene A (CagA) dependent. This response was blocked by pretreatment with GANT61. Anti-PD-L1 treatment of *H*. *pylori* infected huFGOs, co-cultured with autologous patient cytotoxic T lymphocytes and dendritic cells, induced organoid death. *H*. *pylori*-induced PD-L1 expression is mediated by the Shh signaling pathway within the gastric epithelium. Cells infected with *H*. *pylori* that express PD-L1 may be protected from the immune response, creating premalignant lesions progressing to gastric cancer.

## Author summary

Gastric cancer is the 5^th^ most common cancer worldwide and the 3^rd^ most common cause of cancer-related death. *Helicobacter pylori* (*H*. *pylori*) infection is the major risk factor for the development of gastric cancer. Our laboratory has reported that the Sonic Hedgehog (Shh) signaling pathway is an early response of the gastric epithelium to infection and fundamental to the initiation of *H*. *pylori*-induced gastritis. *H*. *pylori* also induces programmed death ligand 1 (PD-L1) expression on gastric epithelial cells, yet the mechanism is unknown. PD-L1 is a protective ligand that is known to suppress the immune system by shutting down T cell effector function. We hypothesized that *H*. *pylori*-induced PD-L1 expression within the gastric epithelium is mediated by the Shh signaling pathway during infection. Moreover, we showed that metaplastic cells may survive chronic inflammation by expressing the immunosuppressive ligand PD-L1 for the persistence of infection and progression of disease to cancer.

## Introduction

*Helicobacter pylori (H*. *pylori)* infects nearly 50% of the world's population and is the number one risk factor for gastric cancer [1]. Albeit a controversial issue, it may be that although *H*. *pylori* infection treated with antibiotics is cleared, once a patient has progressed to a metaplastic phenotype, elimination of the bacteria does not reduce the risk of developing gastric cancer [2]. *H*. *pylori* induces pathogenesis by injecting one key virulence factor cytotoxic associated gene A (CagA) into the gastric epithelial cells [3]. Importantly, CagA stimulates a drastic increase in Sonic Hedgehog (Shh) signaling from parietal cells, a response that is mediated by NFκB signaling [4, 5]. Shh is a gastric morphogen known to initiate gastritis in response to *H*. *pylori* infection [4]. Upon infection *H*. *pylori* induces the secretion of Shh from the acid-secreting parietal cells [4]. Following a sustained increase in Shh secretion and signaling, macrophages are recruited to the infection site [4]. These macrophages secrete IL-1β which inhibits acid secretion causing atrophic gastritis and the atrophy of parietal cells [4, 6]. Overall, Shh signaling plays a fundamental role in the initiation of *H*. *pylori*-induced gastritis [4, 5]. It has also been observed that following *H*. *pylori* infection programmed death ligand 1 (PD-L1) expression on the gastric epithelium is drastically increased [7]. The expression of PD-L1 in human gastric biopsies of infected patients has never been investigated. PD-L1 interacts with programmed death 1 (PD1) on the surface of cytotoxic T lymphocytes (CTLs) rendering CTLs unable to induce apoptosis [8, 9]. Thus, PD-L1 signaling induces cellular proliferation and survival [10, 11].

*H*. *pylori* infection combined with the atrophy of the acid secreting parietal cells leads to the development of spasmolytic polypeptide/Trefoil Factor (TFF) 2-expressing metaplasia (SPEM) [12, 13]. SPEM is the first step in a series of neoplastic changes that occur in the gastric epithelium prior to the development of gastric cancer [14, 15]. In the setting of chronic inflammation and persistent bacterial infection there is the progression of SPEM to intestinal metaplasia and gastric cancer [15]. PD-L1 is a protective ligand that is known to suppress the immune system by shutting down T cell effector function [8, 9]. Here we demonstrate that *H*. *pylori*-induced PD-L1 expression is mediated by Shh signaling as an early epithelial response to infection and a mechanism by which the bacteria evades the immune response. We also demonstrate here that SPEM cells may survive chronic inflammation by expressing the immunosuppressive ligand PD-L1 for the persistence of infection and progression of disease to cancer.

## Results

### Increased PD-L1 expression in *H*. *pylori* Infected FHGOs is mediated by hedgehog signaling

**Fig 1:**
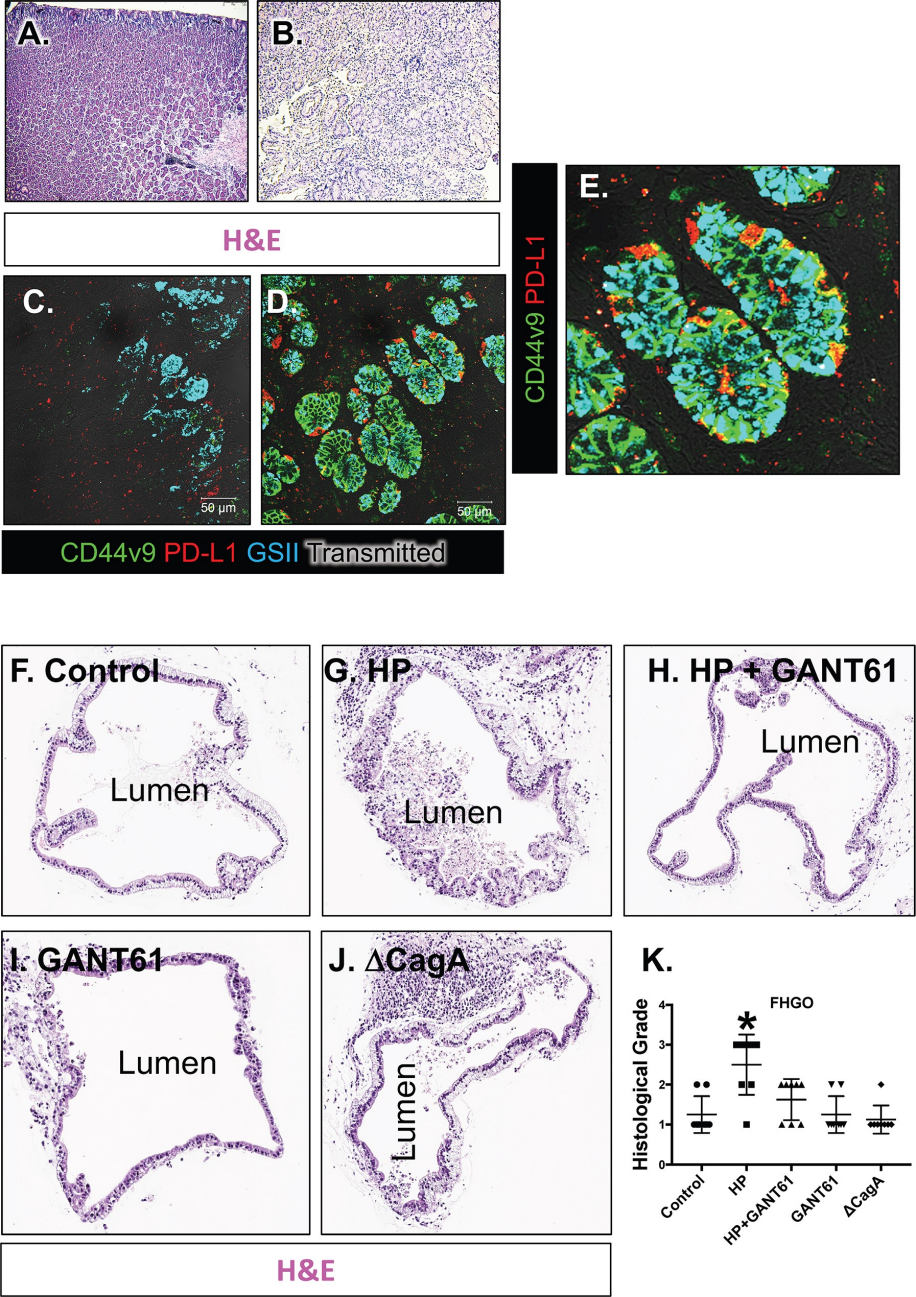
Changes in PD-L1 expression in *H*. *pylori* infected human stomach and histological grade of HGOs. H&E staining of biopsies collected from a **(A)** normal uninfected and **(B)** *H*. *pylori (*HP) infected patient stomach. Immunofluorescence staining for PD-L1 (red) and SPEM marker GSII (cyan) and gastric cancer stem cell marker CD44v9 in **(C)** uninfected and **(D)** HP infected patient stomach. Co-localization of PD-L1 (red) and CD44v9 (green) is seen in **(E).** H&E staining of organoids embedded from **(F)** control, **(G)** HP infected, **(H)** HP+GANT61, and **(I)** GANT61 treated, and **(J)** CagA mutant HP strain (ΔCagA) infected FHGOs. Histological grade assigned to each experimental group from **(K)** FHGOs. *P<0.05 compared to control group by one-way ANOVA, n = 8 individual organoids.

To determine whether *H*. *pylori* induces PD-L1 expression in the stomach, we first collected gastric biopsies from uninfected normal patients (**Fig 1A**), and infected patients that exhibited metaplasia (**Fig 1B**). Compared to the normal control patients (**Fig 1C**), there was an increase in PD-L1 expression in response to *H*. *pylori* infection (**Fig 1D and 1E**). PD-L1 expression within the infected stomach co-localized with SPEM glands that co-expressed Trefoil factor 2 (TFF2) and CD44v9 [16, 17] within the metaplastic epithelium (**Fig 1D and 1E**).

**Fig 2:**
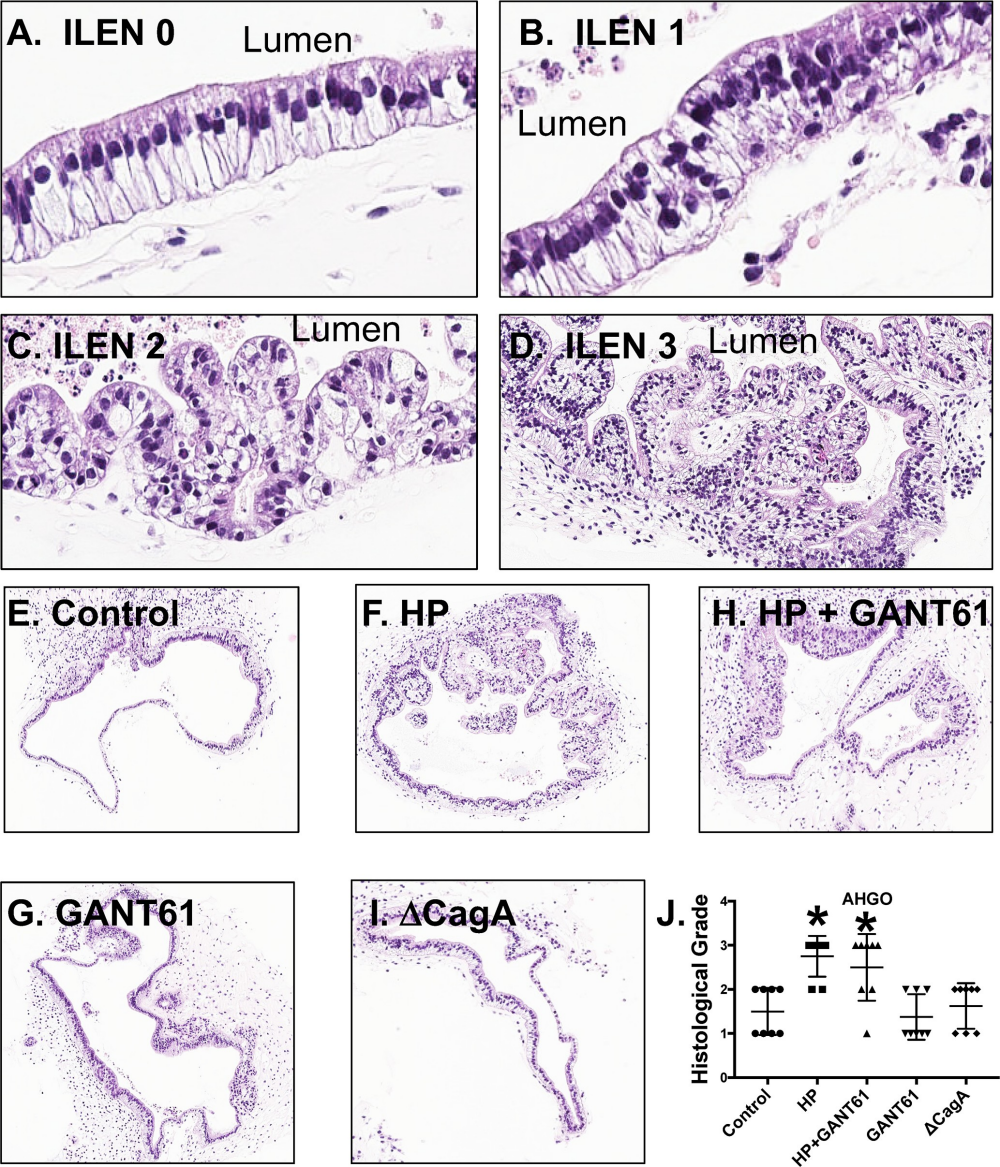
Metaplastic changes in FHGOs and AHGOs infected with *H*. *pylori*. Histological grade was assigned to experimental groups using **I**ntra**l**umenal **E**pithelial **N**eoplasia criteria of **(A)** 0, **(B)** 1, **(C)** 2 or **(D)** 3. H&E staining of organoids embedded from **(E)** control, **(F)** HP infected, **(G)** HP infected and GANT61, and **(H)** GANT61 treated, and **(I)** ΔCagA infected AHGOs. Histological grade assigned to each experimental group from **(J)** AHGOs. *P<0.05 compared to control group by one-way ANOVA, n = 8 individual organoids.

**Fig 3:**
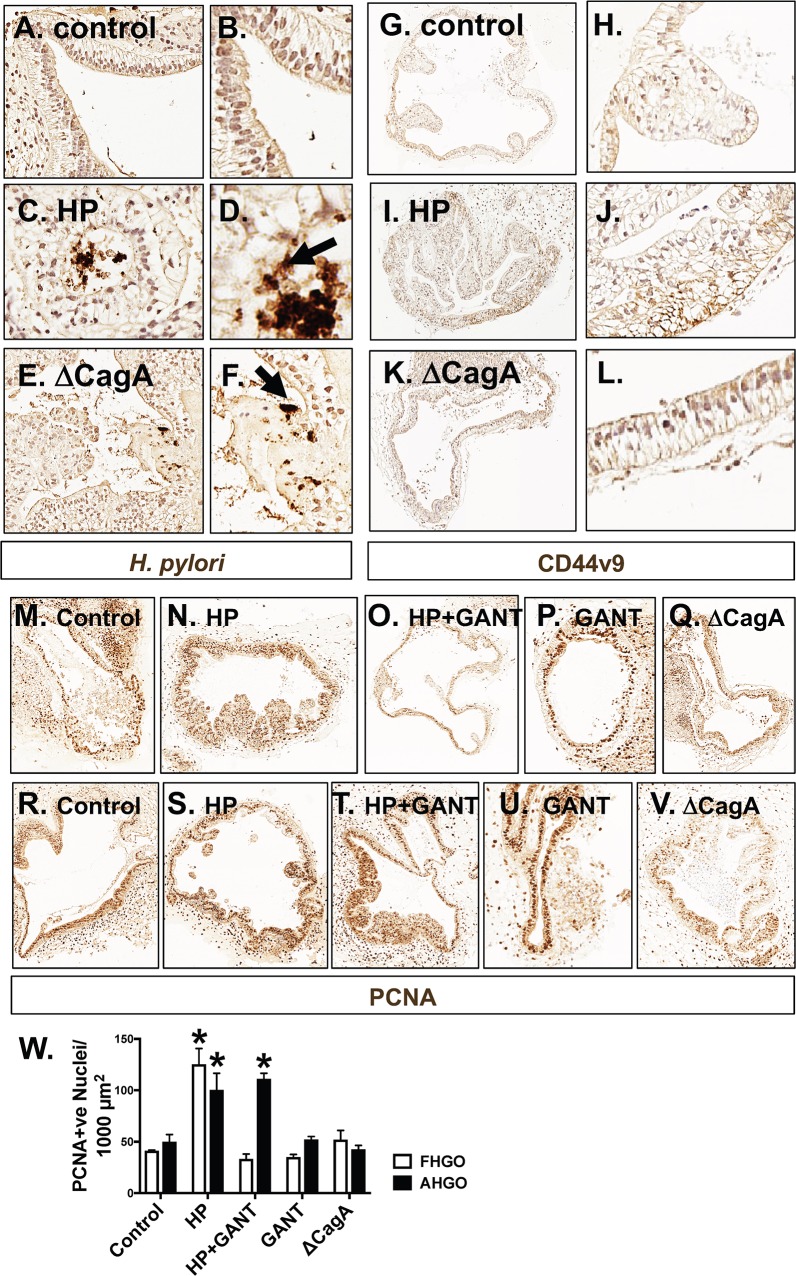
Characterization of *Helicobacter pylori* infected FHGOs and AHGOs. Immunohistochemistry for HP colonization in sections collected from **(A, B)** control magnified **(C, D)** HP and **(E, F)** ΔCagA infected FHGOs. Immunohistochemistry for gastric cancer stem cell marker CD44v9 in sections collected from **(G, H)** control magnified **(I, J)** HP and **(K, L)** ΔCagA infected FHGOs. Immunohistochemistry of PCNA (proliferating cells) in **(M)** control**, (N)** HP infected**, (O)** HP infected and GANT 61 treated**, (P)** GANT61 treated and **(Q)** ΔCagA infected FHGOs. Immunohistochemistry of PCNA in **(R)** control**, (S)** HP infected**, (T)** HP infected and GANT 61 treated**, (U)** GANT61 treated and **(V)** ΔCagA infected AHGOs. **(W)** Quantification of PCNA expressing epithelial cells in FHGOs and AHGOs. *P<0.05 compared to control group by one-way ANOVA, n = 3 area’s averaged for 3 individual organoids.

The effect of *H*. *pylori* infection on the gastric epithelium was then investigated using gastric organoids derived from human induced pluripotent stem cells (HGOs) (**Fig 1F–1K**). PSC-derived HGOs are truly naïve gastric tissue that has never been exposed to any commensal or pathogenic bacteria. In addition, HGOs can be generated into regionally specific gastric organoids that have either fundic or antral epithelium thus allowing us to investigate the unique effects of the two different epithelia. Fundic/corpus (FHGOs) and antral (AHGOs) gastric organoids were infected with *H*. *pylori* for 72 hours. Histological evaluation revealed that compared to control (**Fig 1F**) FHGOs, there was the development of a dysplastic epithelium in response to *H*. *pylori* infection (**Fig 1F and 1K**). Treatment of infected FHGOs with Hedgehog signaling inhibitor GANT61, resulted in the inhibition of the development of dysplasia (**Fig 1H and 1K**). FHGOs infected with a mutant G27 *H*. *pylori* strain bearing a CagA deletion (ΔCagA) did not exhibit that same morphological changes in the epithelium as that observed with the wild type G27 strain (**Fig 1J and 1K**) despite colonization of that both bacterial strains within the organoids (**Fig 3A–3F**). While *H*. *pylori* infection also induced dysplasia in AHGOs, in contrast to FHGOs, GANT61 treatment did not inhibit this response (**Fig 2E–2J**). **Fig 2A–2D** are representative images of the grading scale used to score the histology of infected HGOs. To identify whether the morphological changes observed in the HGOs in response to *H*. *pylori* infection were metaplastic changes, we immunostained sections prepared from organoids for gastric cancer stem cell and SPEM marker CD44v9 (**Fig 3G–3L**). While CD44v9 was not expressed in either the control or ΔCagA infected FHGOs (**Fig 2G, 2H, 2K and 2L**), there was a robust induction of this marker in FHGOs infected with *H*. *pylori* (**Fig 3I and 3J**) and this correlated with a significant increase in epithelial cell proliferation **(Fig 3M–3Q and 3W**). The proliferative response was abrogated with GANT61 treatment of FHGOs (**Fig 3M–3Q and 3W**). A similar response was observed in AHGOs infected with *H*. *pylori*, although GANT61 did not block the proliferation as seen in the FHGOs (**Fig 3R–3V and 3W**).

### *H*. *pylori* infection induces sonic hedgehog (Shh) expression in FHGO with acid-secreting parietal cells

**Fig 4:**
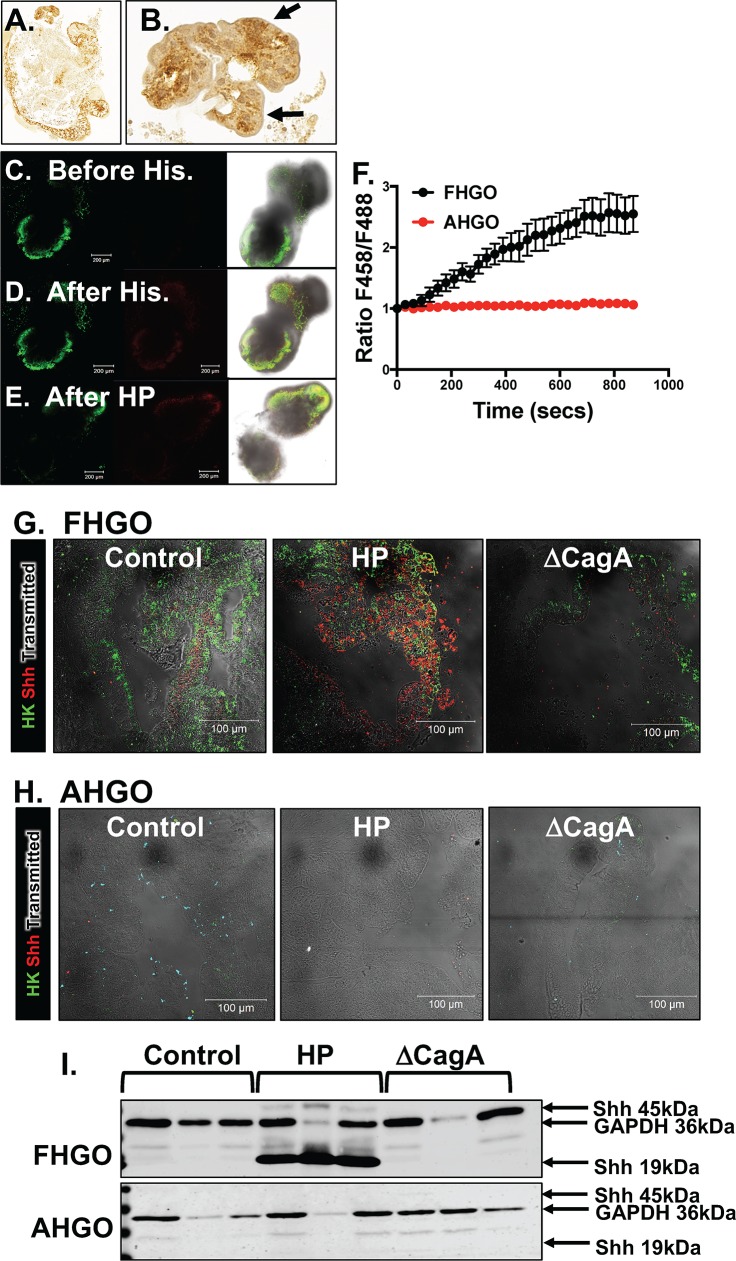
Phenotypic characterization of parietal cells in FHGOs and AHGOs. **(A)** Immunohistochemistry of H+/K+ ATPase in sections collected from FHGOs. Higher magnification shown in **(B).** Acridine orange accumulation assay of FHGOs **(C)** before, **(D)** after histamine stimulation, and **(E)** 24 hours after HP infection**. (F)** Quantification of the ratio shift in F458 (red)/F488 (green) in treated FHGOs and AHGOs. *P<0.05 compared to control group by Student’s T test, n = 3 individual cells. Immunofluorescence staining of sections collected from **(G)** FHGOs and **(H)** AHGOs for the H+/K+ ATPase (green) or Shh (red)**. (I)** Western blots for the expression of GAPDH and Shh in control, and HP and ΔCagA infected FHGOs and AHGOs.

Acridine Orange is a dye known to show green fluorescence (F488) at a neutral pH and a shift in the fluorescent spectrum to red (F458) when it accumulates in the acidic organelles, as the secretory canaliculus of parietal cells [18]. Immunohistochemical staining revealed the clear presence of H^+^,K^+^-ATPase positive parietal cells within the epithelium of FHGOs (**Fig 4A and 4B**). Importantly, in response to histamine, Acridine Orange accumulated in cell vesicles as indicated by the increase in the shift in red fluorescence and increase in the ratio of F458 (red)/F488 (green) (**Fig 4C, 4D and 4F**). AHGOs treated with histamine did not exhibit accumulation of Acridine Orange as documented by a lack in an increase in F458/F488 ratio (**Fig 4K**). FHGOs infected with *H*. *pylori* for 24 hours also exhibited areas of acidic accumulation within the epithelium (**Fig 4E**).

Studies have demonstrated that Sonic Hedgehog (Shh) is found within the gastric parietal cells and processed from a 45kDa to a 19kDa bioactive protein via a mechanism that is acid- and protease-dependent [19–21]. Supported by previous findings, consistent with the expression and secretion of acid within FHGOs, there was a significant increase in the expression of Shh in response to *H*. *pylori*, that was not observed in infected AHGOs that were devoid of parietal cells (**Fig 4G–4I**). The response was CagA dependent (**Fig 4G–4I**). Collectively, these data demonstrate that Shh expression is induced in acid-secreting FHGOs by *H*. *pylori* infection.

### Increased PD-L1 expression in response to *H*. *pylori* infection is localized to the fundus/corpus and mediated by hedgehog signaling

**Fig 5:**
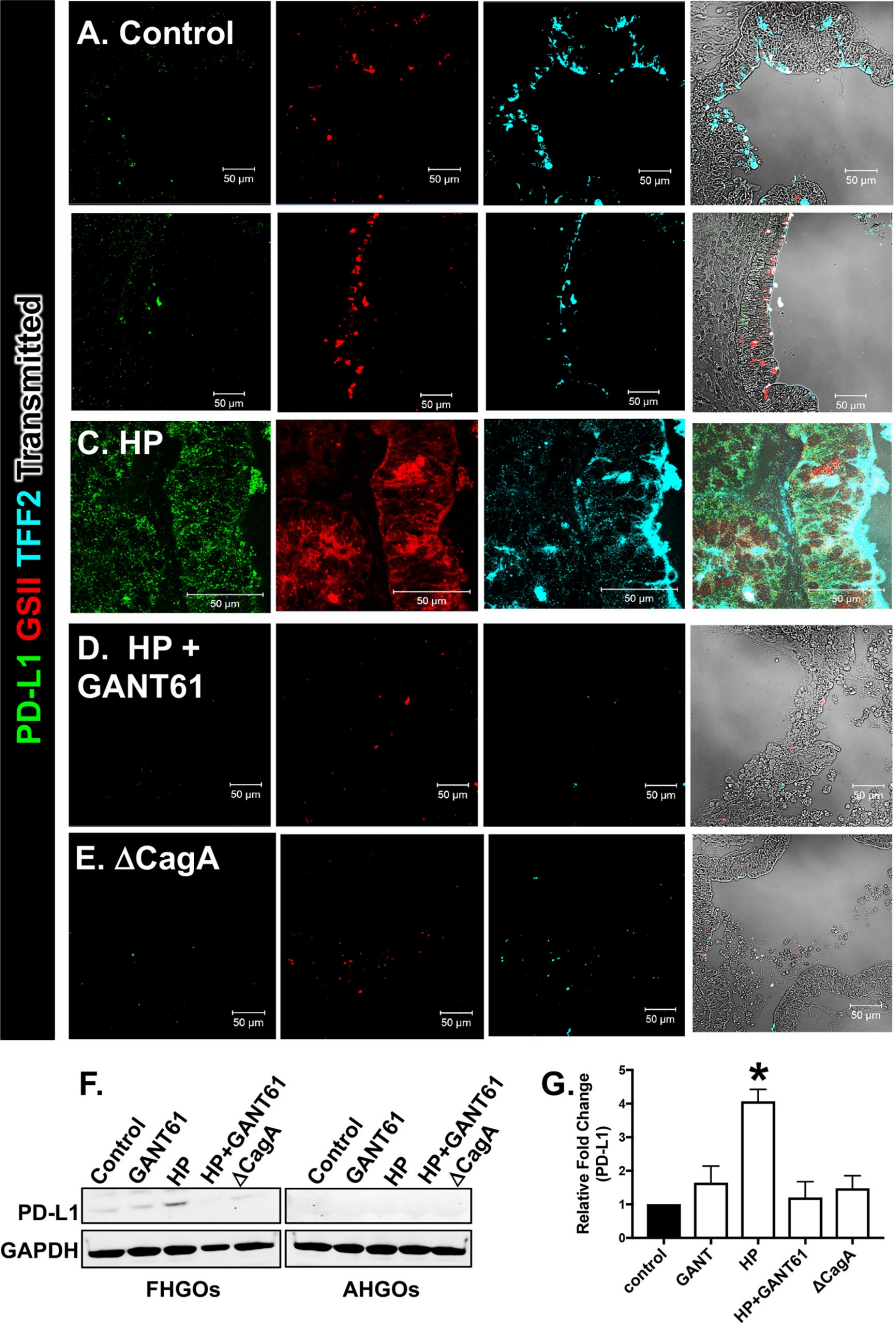
Changes in PD-L1 expression in FHGOs and AHGOs infected with *H*. *pylori*. Immunofluorescence staining for PD-L1 (green), GSII (red) and TFF2 (blue) in **(A)** uninfected control, **(B)** GANT61 treated, **(C)** *H*. *pylori* (HP) infected, **(D)** HP and GANT61 treated and **(E)** ΔCagA infected FHGOs. **(F)** Western blot analysis for the expression of PD-L1 using whole cell lysate collected from each treatment group of the FHGOs and AHGOs. **(G)** Western blots were quantified using ImageJ. *P<0.05 compared to control groups by one-way ANOVA, n = 3 individual organoid experiments.

Immunofluorescence staining and western blot analysis of FHGOs for the expression of PD-L1 and co-expression of SPEM markers TFF2 and *Griffonia Simplicifolia II* (GSII) revealed increased PD-L1 expression within metaplastic glands of infected organoids (**Fig 5C and 5F**). Treatment of infected FHGOs with GANT61 inhibited the PD-L1 expression that was triggered in response to *H*. *pylori* (**Fig 5D and 5F**), when compared to control (**Fig 5A and 5F**) and GANT61 (minus *H*. *pylori*) (**Fig 5B, F**) treated groups. Organoids infected with the G27 *H*. *pylori* strain that expressed a deletion of CagA (ΔCagA) did not differ from the controls with regards to PD-L1 expression (**Fig 5E and 5F**). In contrast to FHGOs, *H*. *pylori* infection did not induce PD-L1 expression as assessed by and western blot (**Fig 5F**).

**Fig 6:**
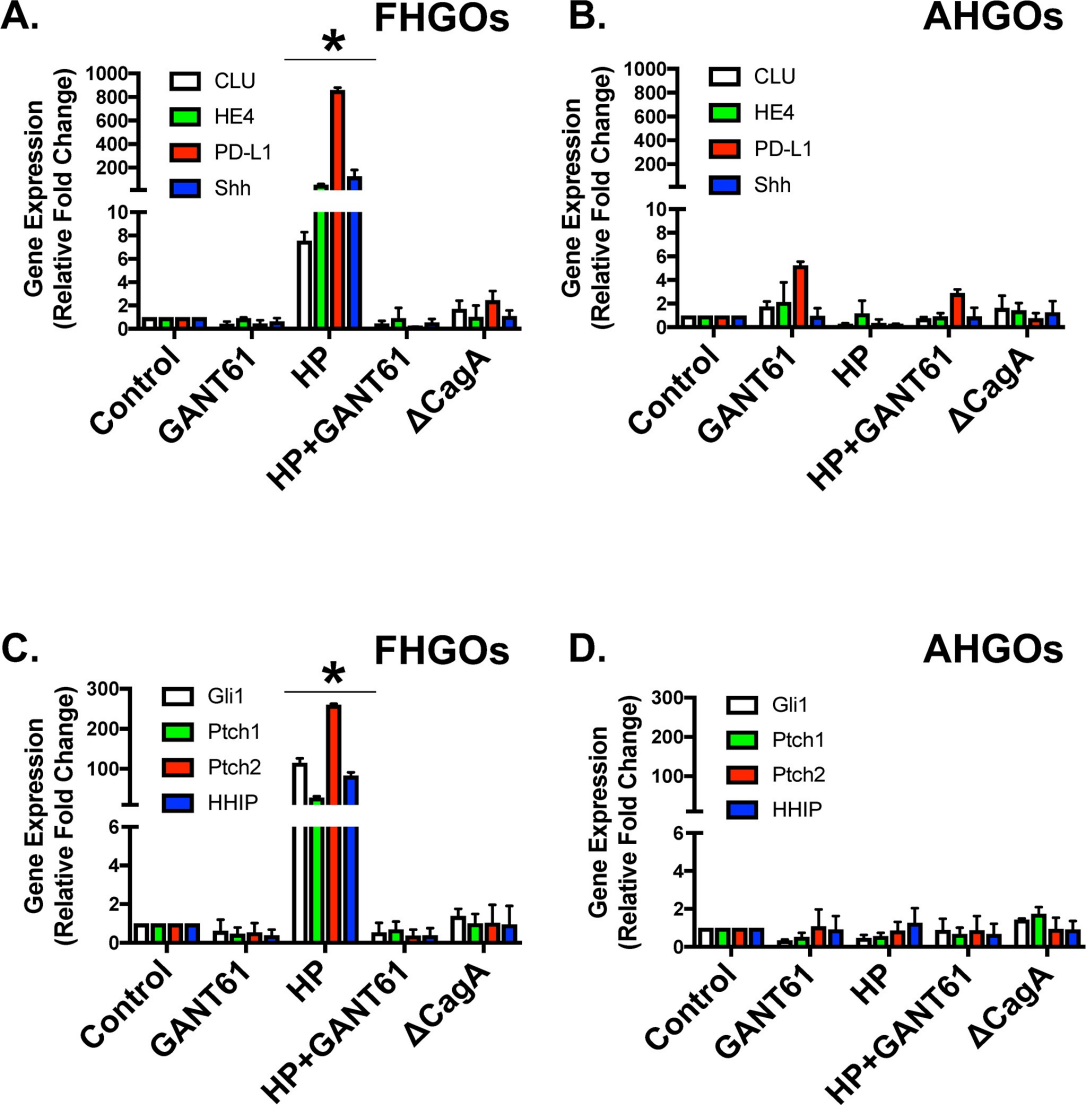
Changes in PD-L1, Hedgehog signaling and markers of SPEM in FHGOs and AHGOs infected with *H*. *pylori*. Quantitative RT-PCR analysis for the expression of PD-L1, Shh and SPEM markers clusterin (CLU) and human epididymis 4 (HE4) performed using RNA collected from each experimental group of the **(A)** FHGOs and **(B)** AHGOs. Quantitative RT-PCR analysis for the expression of canonical Hedgehog signaling genes Gli1, Ptch 1, Ptch 2 and Hedgehog Interacting Protein (HHIP) was performed using RNA collected from each experimental group of the **(C)** FHGOs and **(D)** AHGOs. *P<0.05 compared to control group by two-way ANOVA, n = 3 individual organoid experiments.

Consistent with changes in protein expression, quantitative RT-PCR data showed a significant increase in PD-L1, Shh and SPEM markers clusterin (CLU) and Human Epididymis Protein 4 (HE4) gene expression specifically within the FHGO epithelium (**Fig 6A**), when compared to AHGOs (**Fig 6B**). Hedgehog signals are regulated based on the positive feedback loop via GLI1 and negative feedback loop via patched 1 (PTCH1), patched 2 (PTCH2), and hedgehog interacting protein (HHIP) [22]. We also observed a significant increase in canonical Hedgehog signaling within the FHGO epithelium (**Fig 6C**). A response that was not observed in the AHGOs (**Fig 6D**). Collectively, these data demonstrate that *H*. *pylori*-induced PD-L1 is localized to the fundic/corpus epithelium, and this response is mediated by Hedgehog signaling.

### *H*. *pylori* infection induces PD-L1 expression in a human-derived gastric epithelial monolayer culture

**Fig 7:**
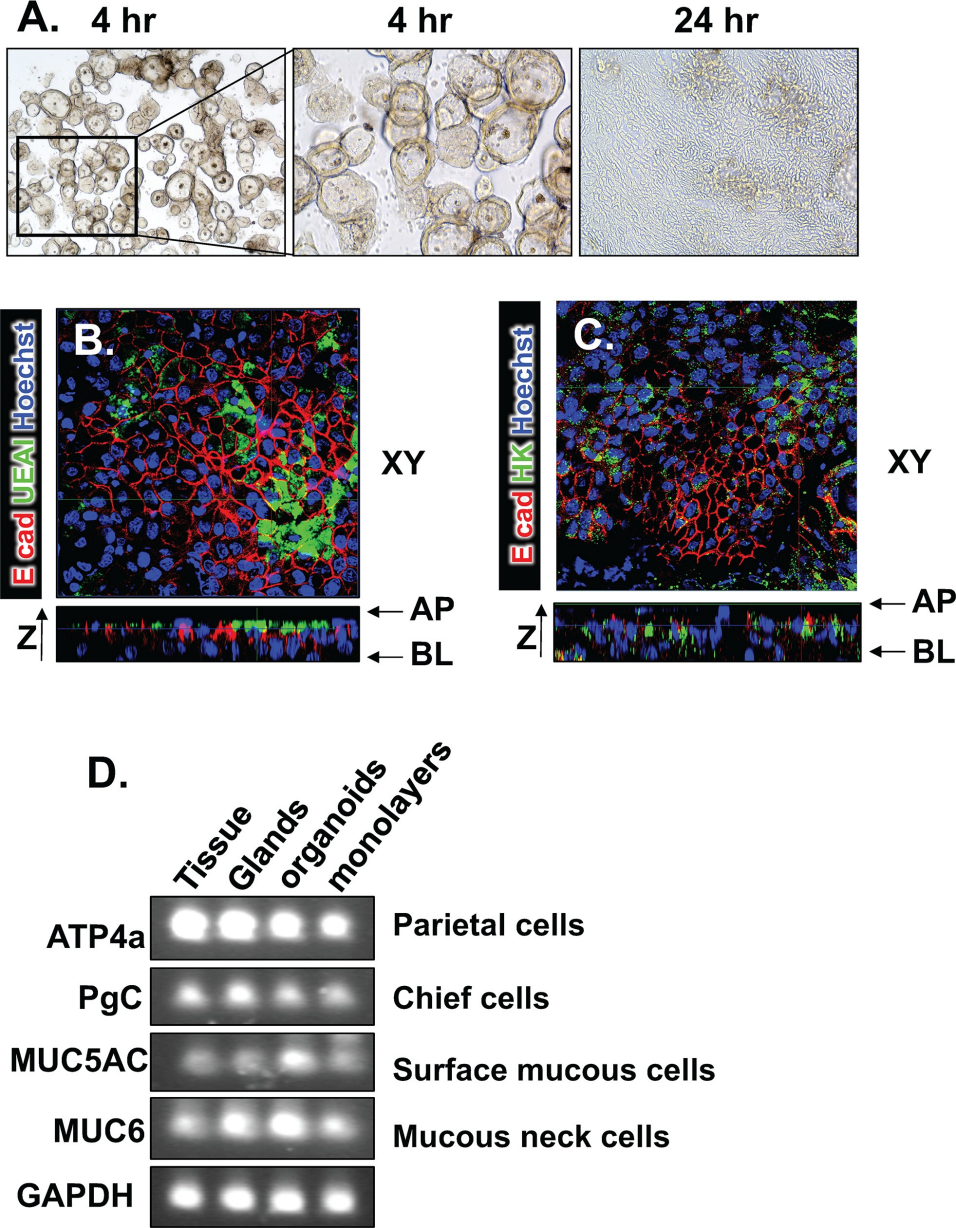
Generation of human-derived gastric epithelial monolayers. **(A)** Light micrographs of organoids transferred to monolayers 48 hours after culture. **(B)** Immunofluorescence of monolayers showing apical expression of surface mucous cell marker UEA1 (green), E-cadherin (E cad, red) and Hoechst (blue). **(C)** Immunofluorescence of monolayers showing expression of parietal cell marker H+,K+-ATPase (HK, green), E cad (red) and Hoechst (blue). **(D)** DNA gel from RT-PCR using RNA collected from gastric tissue, glands, organoids and monolayers.

To identify the mechanism by which *H*. *pylori* induces PD-L1 within the human gastric corpus epithelium, we developed a 2D/monolayer culture of *H*. *pylori* infection using human-derived gastric organoids. We first established fundic organoids derived from human stomachs (huFGO). After 4 days of culture, huFGOs were transferred into 2D dense planar cultures of polarized epithelial cells according to a modification to a published protocol (**Fig 7A**) [23]. Forty eight hours after culture, membranes were collected and immunostained for surface mucous cell marker *Ulex Europeus I* (UEAI) to demonstrate apical expression of this pit cell marker in the polarized gastric cultures (**Fig 7B**) and parietal cell specific H^+^,K^+^-ATPase (**Fig 7C**). The monolayers expressed all major mature gastric cell lineages (**Fig 7D**).

**Fig 8:**
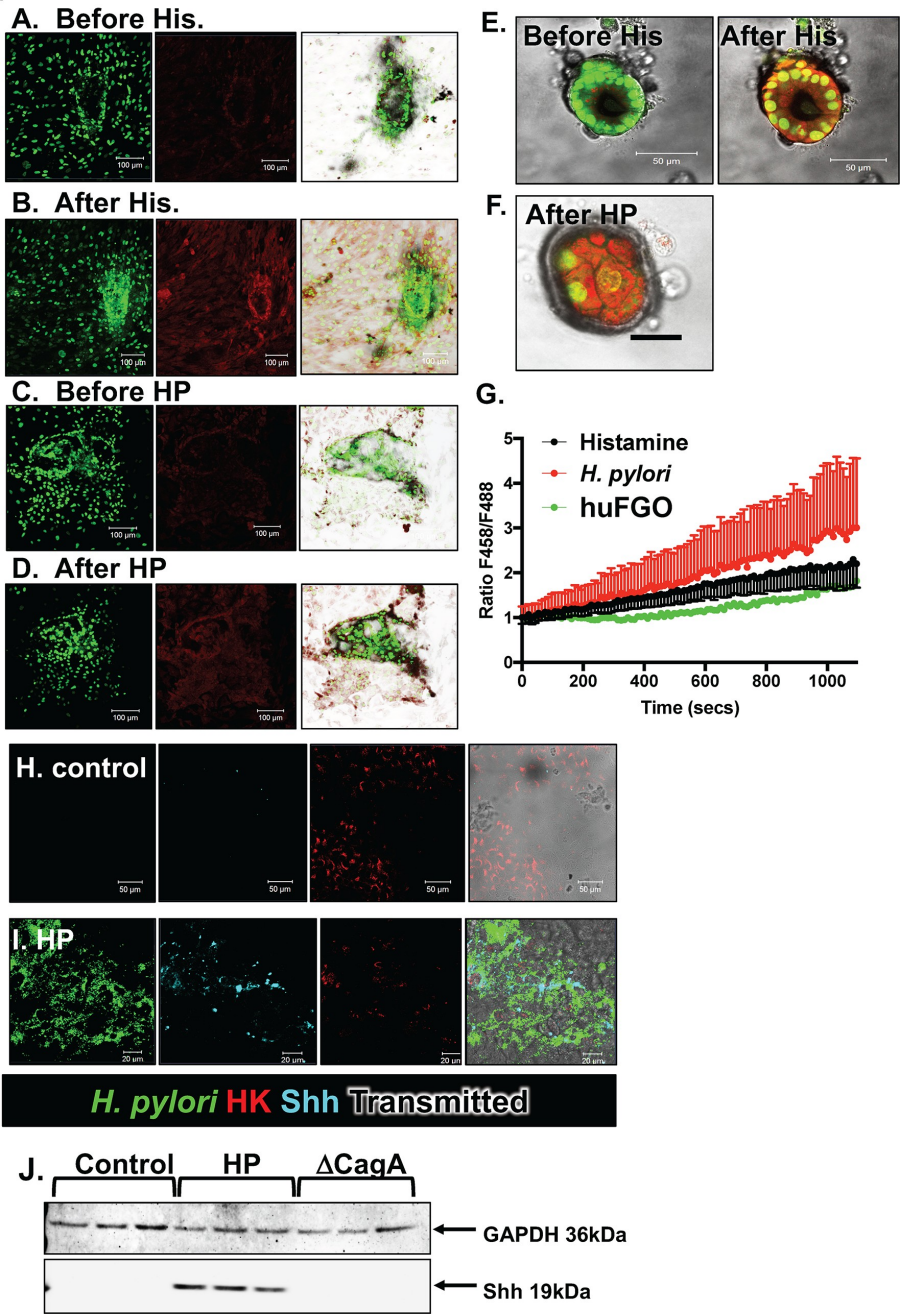
Phenotypic characterization of parietal cells in gastric epithelial monolayers and 3D gastric organoids. Acridine orange accumulation in of gastric epithelial monolayers **(A)** before, and **(B)** after histamine stimulation, **(C)** before and **(D)** after HP infection. Acridine orange accumulation of 3D gastric organoids **(E)** before and after histamine stimulation, and **(F)** after a 24 hour HP infection**. (G)** Quantification of the ratio shift in F458 (red)/F488 (green) in treated gastric epithelial monolayers during treatment (Histamine) or HP infection (*H*. *pylori*), and during histamine treatment of 3D gastric organoids (huFGO). *P<0.05 compared to control group by one-way ANOVA, n = 3 individual cells. Immunofluorescence staining for *H*. *pylori* (green), H^+^/K^+^ ATPase positive parietal cells (red) and Shh (cyan) in **(H)** control, or **(I)** HP infected gastric epithelial monolayers. **(J)** Western blots for the expression of GAPDH and Shh in control, HP and ΔCagA infected gastric epithelial monolayers.

As discussed, Acridine Orange is a dye that accumulates in the acidic organelles such as the secretory canaliculus of parietal cells leading to a fluorescence shift from green to red [18]. As observed in FHGOs, in response to histamine, Acridine Orange accumulated in acidic cell vesicles within gastric epithelial monolayers, and thus leading to the increase in a shift in red fluorescence and increase in the ratio of F458 (red)/F488 (green) (**Fig 8A, 8B and 8G**). A similar response was observed in monolayers infected with *H*. *pylori* (**Fig 8C, 8D and 8G**). Acid secretion was also induced in human-derived gastric organoids (huFGOs) in response to histamine (**Fig 8E and 8G**). The accumulation of Acridine Orange was also observed within resident parietal cells of huFGOs in response to a 24 hour *H*. *pylori* infection (**Fig 8F**). Stimulation of acid secretion in response to *H*. *pylori* infection, correlated with increased Shh expression within H^+^,K^+^-ATPase positive parietal cells (**Fig 8H–8J**). The induction of Shh was CagA dependent (**Fig 8J**).

**Fig 9:**
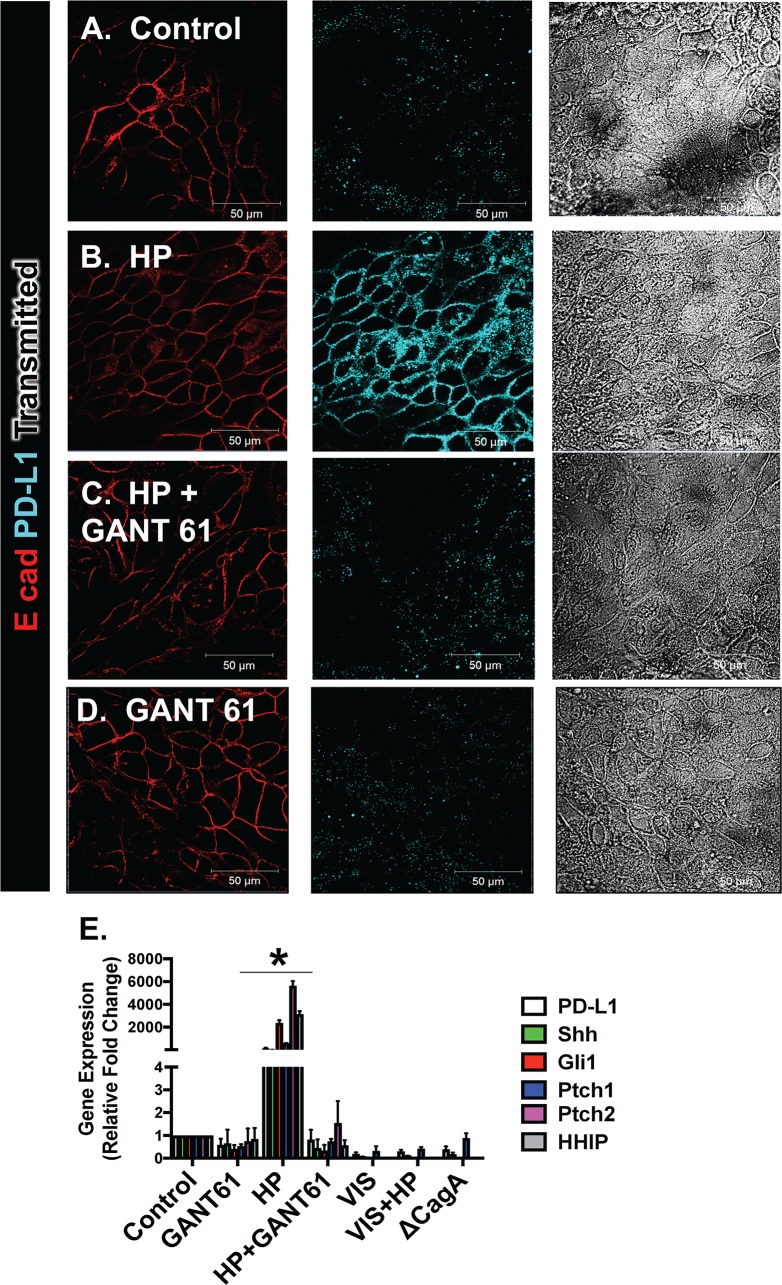
Changes in PD-L1 expression in response to *H*. *pylori* using human-derived gastric epithelial cell monolayers. Immunofluorescence stain of gastric epithelial monolayers collected from **(A)** control, **(B)** *H*. *pylori* (HP), **(C)** HP + GANT61 treated and **(D)** GANT61 treated monolayers for E cadherin (E cad, red) and PD-L1 (blue). **(E)** Quantitative RT-PCR analysis for the expression of PD-L1, Shh and canonical Hedgehog signaling genes Gli1, Ptch 1, Ptch 2 and Hedgehog Interacting Protein (HHIP) was performed using RNA collected from each experimental group. *P<0.05 compared to control group by two-way ANOVA, n = 3 individual organoid experiments.

Human gastric-derived monolayer cultures were infected with *H*. *pylori* with or without pretreatment with Hedgehog signaling Gli inhibitor GANT 61 (**Fig 9A–9D**). We observed an increase in PD-L1 membrane-specific expression following *H*. *pylori* infection (**Fig 9B**) that was blocked with GANT 61 treatment (**Fig 9C**). Quantitative RT-PCR confirmed the significant induction of PD-L1 and Shh expression in response to *H*. *pylori* infection, and this response was mediated by canonical Hedgehog signaling (**Fig 9E**). This response was blocked by a second Hedgehog inhibitor vismodegib (VIS), and appeared to by CagA dependent (**Fig 9E**). Thus, our studies further demonstrate a role of Hedgehog signaling as a mediator of *H*. *pylori*-induced PD-L1 expression during early infection.

**Fig 10:**
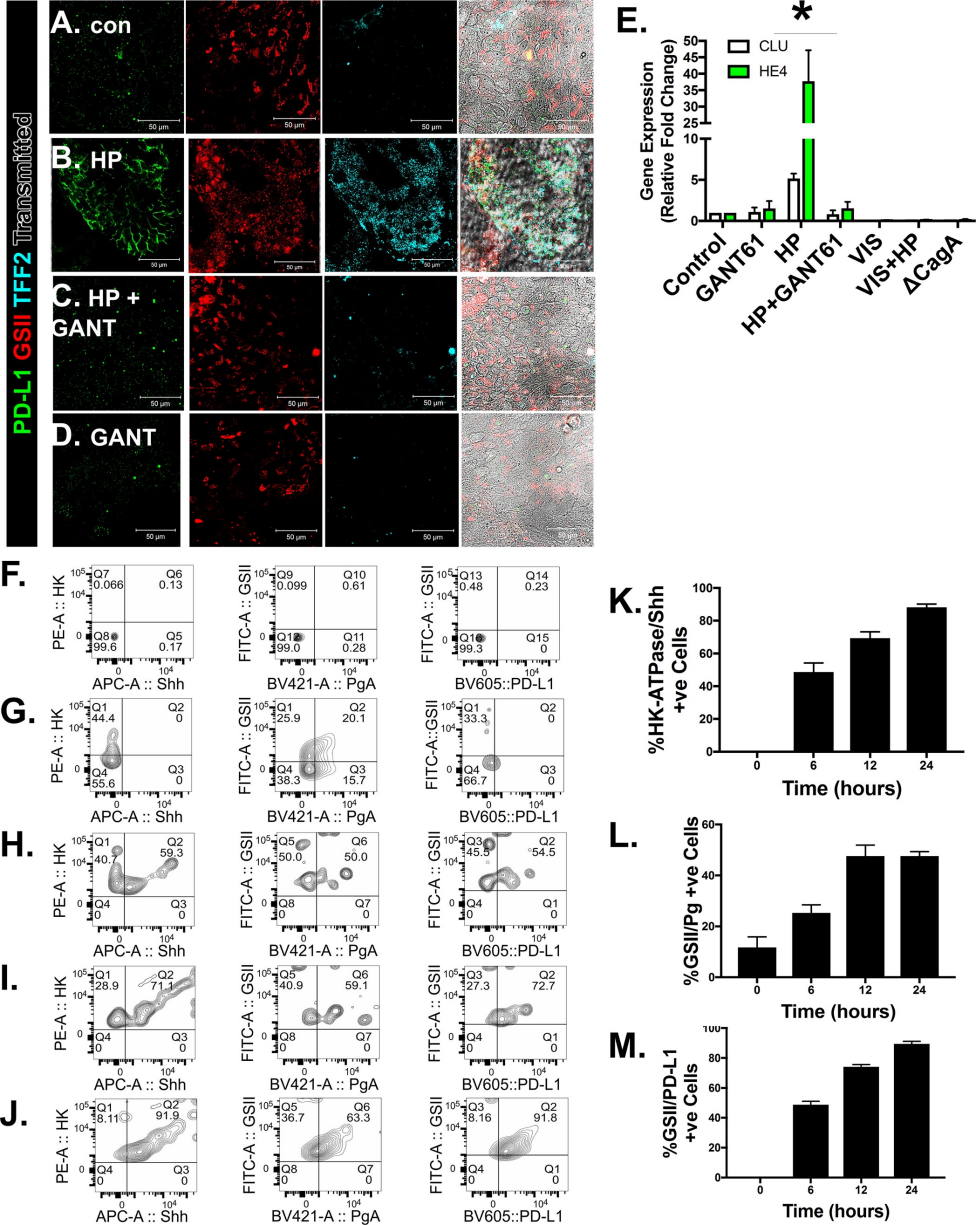
Changes in SPEM markers, and Shh in response to *H*. *pylori* infection using human-derived gastric epithelial cell monolayers. Immunofluorescence stain for PD-L1 (green), GSII (red) and TFF2 (blue) of **(A)** control, **(B)** *H*. *pylori* (HP) infected, **(C)** HP+GANT61 treated, and **(D)** GANT61 treated monolayers. **(E)** Quantitative RT-PCR analysis for the expression of SPEM markers CLU and HE4 was performed using RNA collected from each experimental group. *P<0.05 compared to control group by two-way ANOVA, n = 3 individual organoid experiments. Flow cytometric analysis of cells co-expressing Shh and the H^+^/K^+^ ATPase, the mucus neck cell marker GSII and Pepsinogen A (PgA), or GSII and PD-L1 in **(F)** the unstained population, and **(G)** 0, **(H)** 6**, (I)** 12, and **(J)** 24 hours post HP infection. Quantification of the percent of **(K)** H+/K+ ATPase positive cells that co-express SHH, **(L)** GSII positive cells that co-express PgA, and **(M)** GSII positive cells that co-express PD-L1, 0, 6, 12 and 24 hours post-infection with HP. *P<0.05 compared to control group by one-way ANOVA, n = 3 individual organoid experiments.

**Fig 10** demonstrates that the increase in PD-L1 expression in response to *H*. *pylori* infection was localized to GSII/TFF2 co-expressing cells within the monolayers (**Fig 10B**). These data are consistent with the presence of SPEM markers as confirmed by the increase in CLU and HE4 in response to infection (**Fig 6E**). Interestingly, treatment with GANT61 or VIS not only inhibited PD-L1 expression but also the increase in SPEM markers CLU and HE4 (**Fig 10E**), suggesting a role of Hedgehog signaling in the emergence of metaplasia.

To identify the cellular origin of Shh and PD-L1 expression in response to *H*. *pylori* infection, monolayers were infected with the bacteria for a period of 0 to 24 hours, harvested and analyzed by flow cytometry using markers specific for parietal (H+,K+-ATPase), mucous neck (GSII) and chief (PgA) cells (**Fig 10F–10M**). Compared to the unstained controls (**Fig 10F**), at baseline (0 hours) there was no expression of Shh localized within parietal cells (**Fig 10G and 10K**). However, over a period of a 24 hour infection Shh expression was induced within parietal cells (**Fig 10H–10K**). *H*. *pylori* infection also induced increased GSII/PgA co-expressing cells (**Fig 10G–10J and 10L**). As the mucous neck cells (GSII-expressing) migrate toward the base of the gastric gland, these cells differentiate into the zymogen/chief cells (PgA-expressing) [24]. Importantly, expansion of GSII/PgA co-expressing transitional cells is indicative of the development of SPEM [13, 25]. In addition, PD-L1 was induced within GSII-expressing cells in response to *H*. *pylori* infection (**Fig 10G–10J and 10M**). These data suggest that PD-L1 may be induced specifically within SPEM glands in response to *H*. *pylori* infection.

### *H*. *pylori* infection induces cytotoxic T lymphocyte activation in a human-derived organoid/immune cell co-culture

**Fig 11:**
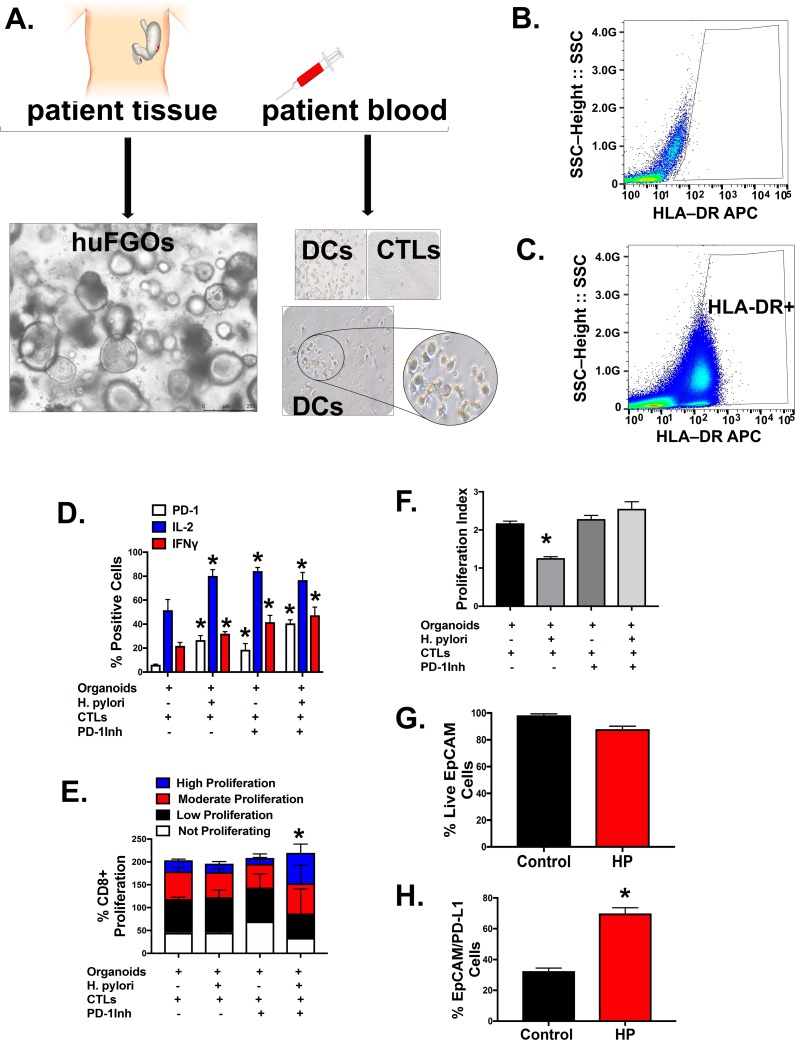
Organoid/dendritic cell and cytotoxic T lymphocyte co-cultures. **(A)** Schematic diagram demonstrating the co-culture of human-derived gastric organoids (huFGOs) and autologous dendritic cells (DCs) and cytotoxic T lymphocytes (CTLs). Representative flow cytometric dot plots of **(B)** unstained control sample, and **(C)** HLA-DR^high^ dendritic cells. **(D)** Quantitative flow cytometric analysis for the percentage of live CD8+ve CTLs extracted from co-cultures expressing PD-1, IL-2 and IFNγ. *P<0.05 compared to control group by two-way ANOVA, n = 3 individual organoid experiments. **(E)** Percentage of CD8+ non-proliferating, low, moderately and high proliferating cells determined by CFSE staining *P<0.05 compared to control group by two-way ANOVA, n = 3 individual organoid experiments, and **(F)** proliferative index. Quantitative flow cytometric analysis of control or HP infected **(G)** % live EpCam positive cells, **(H)** %EpCam Positive cells expressing PD-L1. *P<0.05 compared to control group by one-way ANOVA, n = 3 individual organoid experiments.

PD-L1 interacts with programmed death 1 (PD1) on the surface of cytotoxic T lymphocytes (CTLs) rendering them unable to induce apoptosis [9, 26]. PD-L1 signaling induces cellular proliferation and survival [26]. To study PD-L1/PD-1 interactions between the gastric epithelium and the host's immune response during *H*. *pylori* infection, we developed an organoid/immune cell co-culture system (**Fig 11A**). We obtained autologous patient blood from which dendritic cells were cultured and FACS sorted (**Fig 11B and 11C**). From the blood, CTLs were also isolated and cultured together with the patient's own gastric organoids (**Fig 11A**). After the organoid/immune cell co-culture was infected for 72 hours, CTLs were extracted from the culture by a CD8 positive selection kit. These T cells were analyzed for activation and proliferation by flow cytometry and CFSE uptake (**Fig 11D–11F**). Within the co-culture, *H*. *pylori* significantly induced CTLs to express PD-1, IL-2 and IFNγ (**Fig 11D**). While *H*. *pylori* infection resulted in a decrease in CTL proliferation, treatment with PD-1Inh induced high CTL proliferation (**Fig 11E and 11F**). Unselected cells were then immunostained for CD11c (myeloid-derived dendritic cells) and epithelial marker EpCAM. These cells were then FACs sorted and the EpCAM positive cells were collected and analyzed for PD-L1 expression and cell viability (**Fig 11G and 11H**). HuFGOs infected with *H*.*pylori* had a significantly increased population of EpCam positive cells that expressed PD-L1 when compared to control uninfected huFGOs (**Fig 11H**). These data suggest that while bacterial infection results in decreased CTL proliferation, inhibition of PD-L1/PD-1 interactions induced proliferation of CTLs within the co-culture in the presence of *H*. *pylori* infection.

### Treatment of *H*. *pylori* infected co-cultures induces epithelial cell death

**Fig 12:**
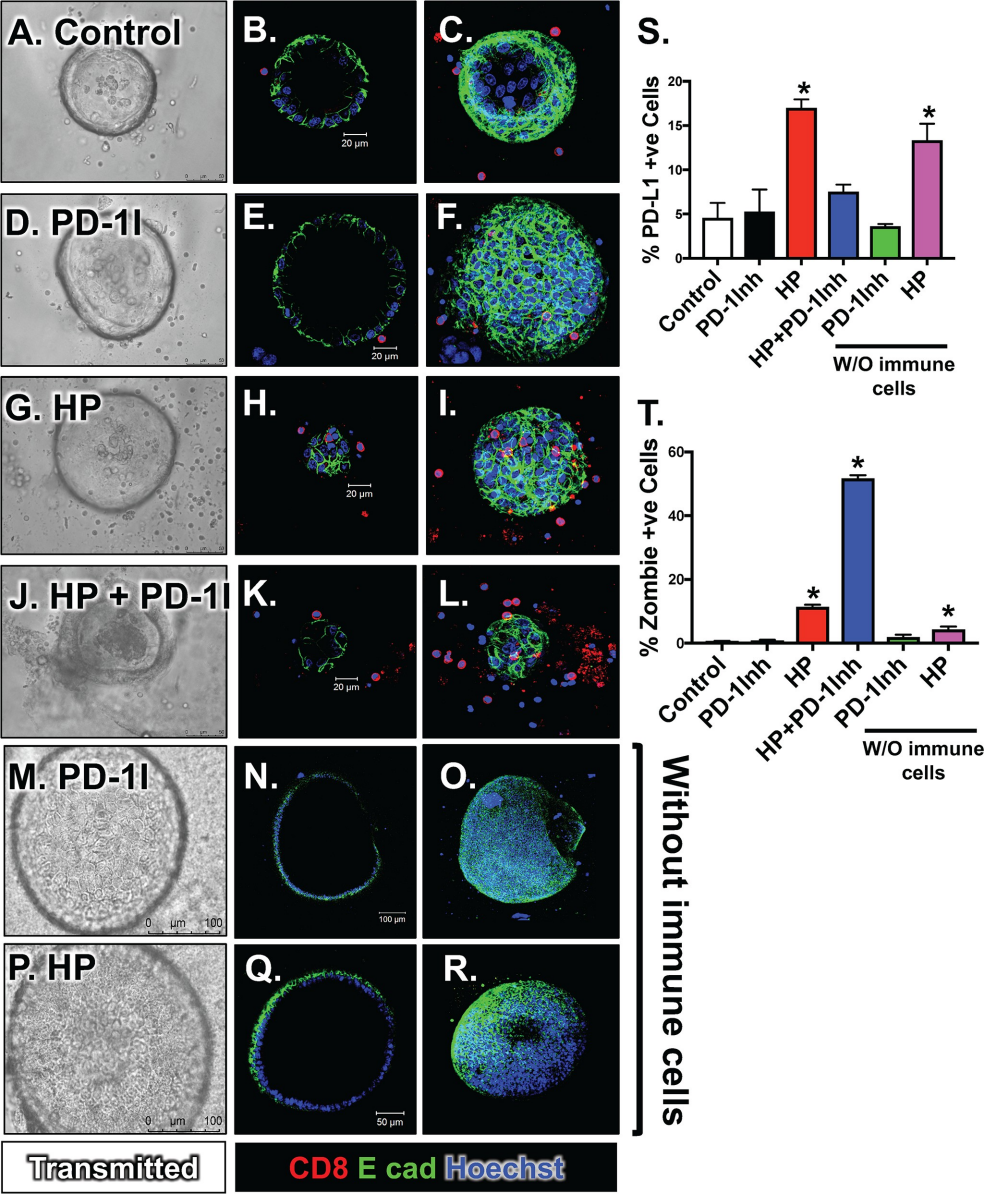
Changes in epithelial cell viability in human-derived organoid/immune cell co-cultures infected with *H*. *pylori*. Light micrographs, immunofluorescence staining for E cadherin (E cad, green) and CD8 (red), and flow cytometric dot plots for percentage of Zombie (dead) PD-L1+ve cells in **(A-C)** control, **(D-F)** PD-1Inh treated, **(G-I)** *H*. *pylori* (HP) infected, and **(J-L)** HP+PD-1Inh treated organoid/immune cell co-cultures, and **(M-O)** PD-1Inh treated organoids and **(P-R)** HP infected organoids. **(S)** Flow cytometric analysis for the percentage of PD-L1 positive cells from control, PD-1I, HP infected and HP+PD-1I treated co-cultures. **(T)** Flow cytometric analysis for the percentage of PD-L1+ve gated Zombie (dead) cells from control, PD-1I, HP infected and HP+PD-1I treated co-cultures. *P<0.05 compared to control group by one-way ANOVA, n = 3 individual organoid experiments.

To investigate the interaction between the infected gastric epithelium and the host's immune response, infected organoids were co-cultured with the patient's DCs and CTLs in the presence and absence of a PD-1Inh and epithelial cell death was measured (**Fig 12**). Compared to the organoid/immune cell co-cultures in the control (**Fig 12A–12C**) or PD-1Inh alone (**Fig 12D–12F**) treatment, *H*. *pylori* infected organoids exhibited a significant increase in cell death with a concomitant increase in PD-L1+ve expressing epithelial cells (**Fig 12G–12I, 12S and 12T**). However, a further significant increase in epithelial cell death was observed in co-cultures treated with PD-1I that was reflected by a decrease in PD-L1+ve expressing epithelial cells (**Fig 12J–12L, 12S and 12T**). PD-1Inh alone without immune cells present had no effect on PD-L1 or organoid viability (**Fig 12M–12O, 12S and 12T**). *H*. *pylori* alone, in the absence of immune cell in culture, continued to have a significant increase in epithelial cell death, however not to the extent as that observed in combination with immune cells and PD-1Inh (**Fig 12T**). Importantly, *H*. *pylori* infection significantly induced PD-L1 expression in the absence of immune cells from the culture (**Fig 12S**). The co-cultures confirm that *H*. *pylori* induces the expression of PD-L1 within the gastric epithelium. In addition, the decreased CTL effector function in response to bacterial infection is inhibited by the PD-1Inh leading to PD-L1 expressing epithelial cell death.

## Discussion

In the current study we show that PD-L1 is induced following initial *H*. *pylori* infection. Previous studies using primary gastric epithelial cells collected from biopsies of patients diagnosed with dyspepsia and gastric cancer cell lines showed an increase in PD-L1 expression following *H*. *pylori* infection [7, 27]. The novelty of this study is the use of gastric organoids derived from patients. To the best of our knowledge we are the first to show induction of PD-L1 expression in human tissue and human derived-organoid models as an early response to bacterial infection. Importantly, induction of PD-L1 expression in gastric organoids and epithelial monolayers was not observed in response to infection with G27 *H*. *pylori* strain bearing a CagA deletion (ΔCagA). The involvement of CagA in *H*. *pylori-*induced PD-L1 expression is significant because CagA is associated with an increased risk of developing gastric cancer [3, 28]. Our data suggests a role of PD-L1 as a potential mechanism by which virulent strains of *H*. *pylori* allow for the persistence of infected gastric epithelial cells.

Shh signaling mediates *H*. pylori-induced PD-L1 expression. Consistent with our findings, we have shown that *H*. *pylori* infection induces an increase in Shh secretion and signaling via a CagA dependent pathway [5]. We further demonstrate that canonical Shh downstream effectors were drastically increased specifically in the fundus/corpus of the stomach following *H*. *pylori* infection. Shh signaling in the epithelium of infected antral differentiated organoids was not observed. An explanation for this observation is data demonstrating that Shh is secreted from the acid-secreting parietal cells within the fundic region of the stomach [29, 30]. Indeed by Acridine Orange accumulation we demonstrate here functional acid-secreting parietal cells with FHGOs, huFGOs and gastric epithelial monolayers. In the presence of GANT 61, a Gli/Hedgehog signaling inhibitor, and vismodegib, that targets the Hedgehog signaling pathway by blocking Ptch and SMO, the expression of canonical SHH downstream effectors decreased. Interestingly, PD-L1 and Shh expression robustly increase in fundic organoids following infection with *H*. *pylori*, a response that was ablated with GANT 61 and vismodegib treatment. Parietal cells in this region secrete Shh which subsequently induces the secretion of acid, normal epithelial cell function and regeneration [29, 31–33]. We advance our initial studies by demonstrating that the release of Shh within the corpus mediates the early induction of PD-L1 expression in response to bacterial infection. In support of our findings, it has been documented that Mycobacteria-responsive Shh signaling within human dendritic cells also mediates PD-L1 expression [34].

In biopsies collected from *H*. *pylori* infected patients, PD-L1 expression co-localized with proteins that classically mark SPEM cells including TFF2 and CD44v9 [16, 17]. Different regions of the stomach respond differently to early transforming factors. For example, individuals most at risk of developing gastric cancer are those in whom the bacteria colonize the corpus (or fundus) of the stomach, when acid secretion is impaired. In contrast, bacterial colonization of the antrum is associated with low levels of inflammation in the corpus, high acid secretion and the development of duodenal ulcer disease [35–37]. Differences in the regional response to *H*. *pylori* infection is evident from our studies. The use of human PSC-derived antral and fundic gastric organoids has allowed us to identify how these unique regions of the human stomach differentially respond to *H*. *pylori* infection.

To identify whether PD-L1 expression protects the epithelium from chronic inflammation, we developed an organoid/autologous immune cell co-culture system. Organoids infected with *H*. *pylori* highly expressed PD-L1 and suppressed CTL proliferation. CTLs are the main pro-apoptotic cell within the gastric cancer microenvironment [38]. When *H*. *pylori* infected organoids were co-cultured with CTLs and treated with a PD-1 inhibitor (PD-1Inh) there was an increase in proliferating CTLs and a decrease in live PD-L1 expressing gastric epithelial cells. Therefore, this suggests that PD-L1 expression was protective to the infected cells. These results are significant because once a patient progresses to a metaplastic state, the eradication of *H*. *pylori* does not decrease the risk of developing gastric cancer [2].

**Fig 13:**
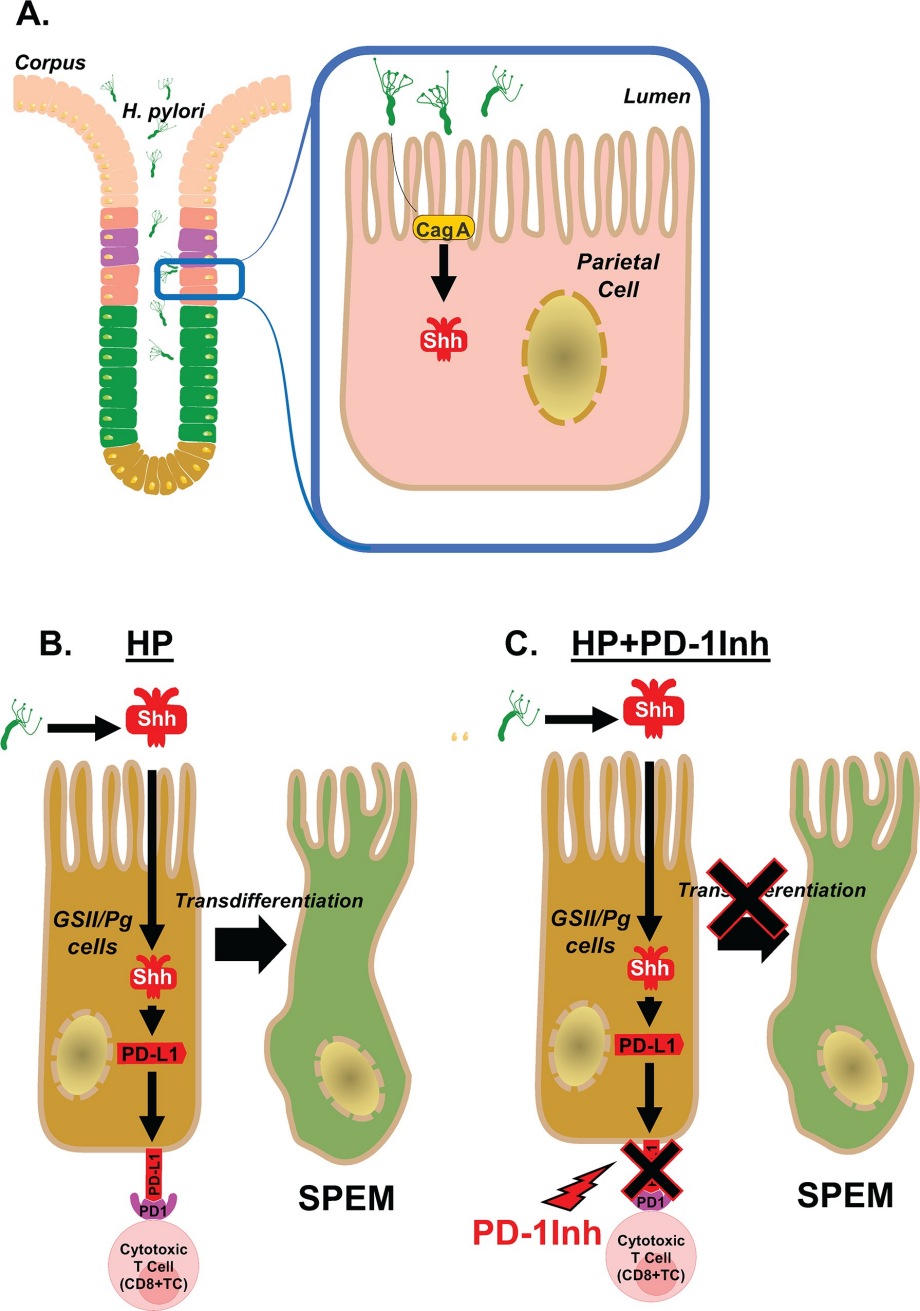
Proposed model of *H*. *pylori* induced PD-L1 expression in the gastric epithelium. **(A)** Shh secretion is induced from the acid secreting parietal cells in response to *H*. *pylori* infection that is driven by a CagA dependent mechanism. **(B)** We propose that Shh induces the expression of PD-L1 on GSII/PgA transdifferentiated cells. PD-L1 then interacts with PD-1 on the surface of CTLs and shuts down the CTL effector function which may lead to the survival of these transdifferentiated metaplastic cells**. (C)** The addition of PD-1Inh blocks the interaction between PD-1 on CTLs and PD-L1 on transdifferentiated/SPEM cells allowing the CTLs to destroy these cells.

PD-L1 expression lasts through to the development of gastric cancer. Up to 69% of all gastric cancers express PD-L1 [39]. Here we present an organoid/immune cell co-culture to model infection with *H*. *pylori* and treatment with immune checkpoint inhibitors. From this study, we proposed that PD-L1, that is induced by parietal cell-derived Shh, may be protective to SPEM cells in the presence of bacterial infection (**Fig 13A and 13B**). When the interaction between PD-1 and PD-L1 is inhibited, activated CTLs may target the SPEM glands (**Fig 13C**). These models could be used to devise a therapy for patients that have progressed to a metaplastic state and would therefore not benefit from eradication of *H*. *pylori*. In addition, this co-culture system could possibly be used to discover new therapies for gastric cancer.

## Materials and methods

### Ethics statement

Human gastric tissue and blood was collected during sleeve gastrectomies were specifically collected for this study with the approval of the Institutional Review Board (IRB protocol number: 2014–0427 Helmrath, Cincinnati Children's Hospital Medical Center and 2015–4869, Zavros, University of Cincinnati). All subjects provided informed written consent. A parent or guardian of any minor participant provided informed consent on their behalf.

### Generation of human induced pluripotent stem cells (iPSCs)

For generation of iPSC263_10 whole blood from a healthy blood donor was obtained from the CCHMC Cell Processing Core, Division of Experimental Hematology and Cancer Biology. Peripheral blood mononuclear cells (PBMCs) were isolated from whole blood using Ficoll centrifugation in SepMate tubes (Stem Cell Technologies). PBMCs were then frozen in cryopreservation media (90% FCS + 10% DMSO) until iPSC generation. PBMCs were thawed and 1-5x10e6 cells were primed for iPSC generation by culture in erythroid expansion media for 8 days (EEM; StemCell Technologies). During priming, 1mL of fresh EEM was added to existing media every 2 days. At the completion of priming (d0), 1x10e6 cells were transduced for 3 h with recombinant VSV-G pseudotyped polycistronic lentiviral particles co-expressing reprogramming factors Oct4, Klf4, Sox2, cMyc and dTomato (Warlich et al., 2011) in the presence of 8ug/mL polybrene. Transduced cells were then plated on 0.1% gelatin-coated dishes containing 2 x 10e4 irradiated MEFs/cm^2^ (GlobalStem) in 2mL EEM. On d2, 1 mL fresh EEM was added to wells. On days 3 and 5, 1 mL hESC media (DMEM:F12 containing 20% knockout serum replacement, 1 mM L-glutamine, 0.1 mM β-mercaptoethanol, 1x non-essential amino acids, and 4ng/mL bFGF) was added to the existing media in each well. Starting on d5, wells underwent a complete daily media change with 2.5 mL hESC media. Putative iPSC colonies were then manually excised and replated in feeder free culture conditions consisting of matrigel (BD BioSciences) and mTeSR1 (Stem Cell Technologies). Lines exhibiting robust proliferation and maintenance of stereotypical human pluripotent stem cell morphology were then expanded and cryopreserved at ~ passage 10 [40, 41]. Donor material for preparation of iPSCs was demonstrated mycoplasma-free using the MycoAlert kit (Lonza; LT07-118). The assay was performed exactly as recommended by the manufacturer and included the use of a positive control (Lonza; LT07-518).

### Generation of organoids from human gastric tissue

Human stomach was digested to glands and embedded into Matrigel^TM^ following a published protocol [16, 42]. Briefly, the epithelium was dissociated from the muscle layer, finely minced and washed in sterile PBS without Ca^2+^ and Mg^2+^ supplemented with 1% Penicillin/Streptomycin. Epithelial tissue was further digested in DMEM/F12 (1263–010, Gibco Life Technologies) containing collagenase A (from *Clostridium histolyticum*, Sigma C9891, 1 mg/ml) and bovine serum albumin (2 mg/ mL) for 15–30 min to liberate glands from tissue. The reaction was stopped using DMEM/F12 (1263–010, Gibco Life Technologies) supplemented with Kanamycin (50 mg/ml) and Amphotericin B (0.25 mg/ml)/Gentamicin (10 mg/ml), and the glands were filtered through sterile gauze and allowed to settle on ice for 10 mins. Glands were washed with PBS supplemented with Kanamycin (50 mg/ml) and Amphotericin B (0.25 mg/ml)/Gentamicin (10 mg/ml) and suspended in Matrigel^TM^. Organoids were plated at a density of 50 μL/well and cultured in 3D human gastric organoid media (DMEM/F12 supplemented with 10 mM HEPES, 1X Glutamax, 1% Pen/Strep, 1X N2, 1X B27, 1 mM N-Acetylcystine, 10 mM Nicotidamide, 50 ng/mL Epidermal Growth Factor (EGF), 100 ng/mL Noggin, 20% R-Spondin Conditioned Media, 50% Wnt Conditioned Media, 200 ng/mL FGF10, 1 nM Gastrin, 10uM Y-27632, Kanamycin (50 mg/ml) and Amphotericin B (0.25 mg/ml)/Gentamicin (10 mg/ml)). Following 6–7 days 3D organoids grew from glands. Following this time 3D organoids were infected with *H*. *pylori* or transferred to 2D organoid monolayers.

### Generation of human-derived gastric epithelial monolayers

Human-derived gastric epithelial monolayers were prepared according to a modified published protocol [23]. Organoids were harvested form Matrigel^TM^ using cold PBS. Organoids were suspended in 2D media containing (DMEM/F12 supplemented with 10% Fetal Calf Serum, 10 mM HEPES, 2 mM GlutaMAX, 1% Pen/Strep, 1X N2, 1X B27, 10 mM Nicotidamide, 50 ng/mL EGF, 10 nM Y-27632, 1 nM Gastrin, 50 mg/ml Kanamycin and 1 μM TGF-βI) and plated onto Matrigel^TM^ coated plates. Briefly, Matrigel^TM^ was diluted tenfold into cell culture grade water and allowed to coat 2 well chamber slides or 12 well plates at 37°C for 1 hour. Excess water was removed from the plate and Matrigel^TM^ coating was allowed to dry for 1 hour at room temperature.

### *Helicobacter pylori* culture and infection

*H*. *pylori* strain G27 [43, 44] and ΔCagA strain, a mutant strain of G27 bearing a CagA deletion (Δ*cagA*::*cat)* [45], were grown on blood agar plates containing a Columbia Agar base (Fisher Scientific) containing 5% horse blood (Colorado Serum Company), 5 μg/ml vancomycin and 10 μg/ml trimethoprim as previously described [4, 5]. HGOs cultured for 32 days and HuFGOs cultured for 7 days were injected with 200 μL of *Brucella* broth containing approximately 2X10^5^ bacteria using a Nanoject II (Drummond) microinjector. Gastric epithelial monolayers cultured for 4 days were infected with 50 μL of DMEM/F12 (1263–010, Gibco Life Technologies) containing 5-8million bacteria.

### Monocyte extraction from whole blood

Whole blood was collected from young sleeve gastrectomy patients (15–21 years old). The Sepmate^TM^ tubes (Stemcell) and Lymphoprep^TM^ (Stemcell) were used to separate out red blood cells and platelets according to manufacturer’s protocol. Briefly, 50 mL Sepmate^TM^ tubes were filled at the bottom with 15 mL of Lymphoprep^TM^. Whole blood was diluted with phosphate-buffered saline containing 2% fetal bovine serum. Diluted whole blood was added to the tube containing Lymphoprep^TM^. The tubes were centrifuged at 1200 *g* for 10 minutes. Following this supernatant was poured into a separate tube. The supernatant was diluted with phosphate-buffered saline containing 2% fetal bovine serum. The supernatant was centrifuged at 300 *g* for 8 minutes. The supernatant was discarded, and the pellet was re-suspended in phosphate-buffered saline containing 2% fetal bovine serum. The pellet was centrifuged at 120 *g* for 10 minutes. The resulting peripheral blood mononuclear cells were cultured in dendritic cell media or put through the negative selection EasySep^TM^ Human CD8+ T cell Enrichment Kit (Stemcell).

### Dendritic cell maturation

PBMCs were matured into dendritic cells using a published protocol [46]. PBMCs are cultured in dendritic base media. Briefly, AIM V cell culture media (Invitrogen) is supplemented with 10% human serum albumin (Gemini BioScience), β-mercaptoethanol (50μM), 1% Penicillin/Streptomycin, 0.1% amphotericin B, 800 U/mL GM-CSF (LifeTechmologies), 500 U/mL IL-4 (LifeTechnologies). After three days cells were fed with dendritic base media. On day 5 immature dendritic cells were fed with dendritic base media supplemented with 5 ng/mL TNF (Life Technologies), 5 ng/mL IL-1β (Life Technologies), 150 ng/mL IL-6 (Life Technologies), and 1 μg/mL prostaglandin E_2_ (PGE_2_; Life Technologies). On day 6 mature dendritic cells were shorted by fluorescence-activated cell sorting (FACs) for the expression of HLA-DR (Biolegend). On day 7 FACs sorted mature dendritic cells were co-cultured with control or *H*. *pylori* huFGOs.

### CD8+ T cell isolation and culture (CTLs)

CD8+ T Cells were extracted from PBMCs isolated from whole blood using the EasySep^TM^ Human CD8+ T cell Enrichment according to manufacturer’s protocol. Briefly, PBMCs were suspended in EasySep^TM^ buffer (Cell Separation Buffer) (Stemcell) in a 14mL round bottom centrifuge tube (Corning). 50 μL/mL of Enrichment Cocktail was added to PBMCs and allowed to incubate at room temperature for 10 minutes. Magnetic particles were mixed by vortexing for 30 seconds. 150 μL/mL of magnetic particles were added to the PBMCs and allowed to incubate for 5 minutes at room temperature. The PBMC cocktail was topped up to 5 mL using EasySep^TM^ Buffer. The PBMC cocktail was added to “The Big Easy” magnet (Stemcell) and allowed to incubate at room temperature for 5 minutes. The CD8+ T cells are the cells that have no bound magnets. These were poured into a fresh 15 mL conical and centrifuged at 1200 rpm for 5 minutes and plated in T cell media containing RPMI 1640 (Invitrogen), 10% fetal calf serum, β-mercaptoethanol (50 μM), 1% Pennecillin/Streptomycin, 1% Insulin-tellurium-selenium (Thermofisher), IL-2 (30 U/mL) (Thermofisher) and IL-7 (0.5 ng/mL) (Thermofisher) [9].

### HuFGO, dendritic cell and CD8+ T cell co-culture

HuFGOs were harvested from Matrigel^TM^ with cold DMEM/F12 and centrifuging the organoid suspension at 400 *g* for 5 minutes. CD8+ T cells were harvested and centrifuged at 300 *g* for 5 minutes. CTLs were suspended in a 5 μM Carboxyfluorescein succinimidyl ester (CFSE) for 20 minutes at 37°C. Following this cells were washed with DPBS and centrifuged at 300 *g* for 5 minutes. CTLs were then incubated in huFGO full media for 10 minutes at 37°C. CTLs were then centrifuged at 300 *g* for 5 minutes. Mature dendritic cells were centrifuged at *300 g* for 5 minutes. HuFGOs, CD8+ T cells and dendritic cells were suspended in Matrigel^TM^ and plated in 4 well plate. HuFGOs were injected with 200 nL of *Brucella* broth containing approximately 2*10^5^ bacteria using a Nanoject II (Drummond) microinjector. One well of uninfected huFGOs co-cultured with CD8+ T Cells and dendritic cells and one well of *H*. *pylori* infected huFGOs co-cultured with CD8+ T Cells and dendritic cells were treated with Nivolumab (A2002, Selleckchem), a PD-1 inhibitor. Cells were co-cultured for 5 days.

### Immunofluorescence

Tissue slides were hydrated with ethanol, xylenes and water. Slides were then blocked with 20% donkey serum at room temperature for one hour and incubated with primary antibodies for PD-L1 (Rat, Novus, 1:100 dilution), TFF2 (Rabbit, 1:200 dilution) or CD44v9 (rat, CosmoBio, 1:1000 dilution), PD-L1 (Rabbit, Novus, 1:100 dilution) or GSII (ThermoFisher, 1:100 dilution) overnight at 4°C. Slides were washed in 0.01% triton x-100 in PBS and treated with a secondary for donkey anti-rat 488, anti-rabbit 647 or donkey anti-rat 488, donkey anti-rabbit 555 and GSII 647 as well (Hoechst 33342, 10 μg/ml, Invitrogen). Media was removed from HGOs, 2D organoid monolayers or huFGO co-cultures with immune cells and 3.7% formaldehyde was added to the organoids for 15 minutes at room temperature. The cultures were washed with PBS and then permeabilized with 0.5% Triton X-100 in DPBS for 20 minutes at room temperature. Blocking was done with 2% normal donkey serum for 1 hour at room temperature. Monolayer cultures were then incubated overnight at 4°C with primary antibodies specific for H+/K+ ATPase (Thermofisher, mouse, 1:1000 dilution) and E-cadherin (R&D, goat, 1:400 dilution). HGOs and monolayers were incubated overnight at 4°C with PD-L1 (Rat, Novus, 1:100 dilution) and TFF2 (Rabbit, 1:100 dilution) or HK (mouse, thermofisher, 1:1000 dilution) and Sonic Hedgehog (Goat, Novus, 1:200 dilution). Monolayers were incubated for 1 hour at room temperature with secondary antibodies donkey anti-mouse 594, UEAI (Sigma, 488), donkey anti-goat 647 or donkey anti-mouse 555 and donkey anti-goat 647 and counter stained with (Hoechst 33342, 10 μg/ml, Invitrogen). Monolayers and HGOs were incubated for 1 hour at room temperature with *Griffonia simplicifolia* (GSII) (Thermofisher, 488, 1:100 dilution), anti-rat 594, anti-rabbit 647 and counter stained with (Hoechst 33342, 10 μg/ml, Invitrogen). huFGOs co-cultured with immune cells were incubated overnight at 4°C with primary antibodies specific for CD8a (Mouse, Novus), CD11c (Rabbit, Novus) and E-cadherin (R&D, Goat). Organoids were then treated with secondary antibodies anti-mouse 594, anti-rabbit 488 or anti-goat 647 and counter stained with (Hoechst 33342, 10 μg/ml, Invitrogen) for 1 hour at room temperature. Organoids were visualized using the Zeiss LSM710.

### Immunohistochemistry

Organoids were fixed in 4% paraformaldehyde for 15 minutes. They were then embedded in paraffin and cut into 5 μM sections. Slides were then deparaffinized and antigen retrieval was done by heating slides for 10 minutes at 100°C in 0.01 M sodium citrate buffer (Antigen Unmasking Solution, Vector Laboratories, Burlingame, CA). Endogenous peroxide activity was then blocked by incubating slides with 0.3% hydrogen peroxide in methanol for 20 minutes. Slides were then incubated with 20% horse serum (PCNA, *Helicobacter pylori* and CD44v9) (ImmPRESS HRP reagent kit, Vector) or 20% goat serum (H+/K+ ATPase). Slides were then incubated with a 1:2000 dilution of PCNA (rabbit, Novus), 1:1000 dilution of H+/K+ ATPase (mouse, Thermofisher) or 1:1000 dilution of CD44v9 (rat, CosmoBio) overnight at 4°C. *Helicobacter pylori* (Rabbit, Ventana) stained slides were incubated with the pre-diluted antibody for 28 minutes at 37°C. Slides were then biotinylated with an IgG secondary antibody for either rabbit, mouse or rat for 30 minutes at room temperature. Finally slides were incubated with ABC reagent (Vectastain ABC kit; Vector Laboratories, Burlingame, CA) for 30 minutes at room temperature. The color of each set of slides was then developed with 3,3’-diaminobenzidine (DAB) from the DAB Substrate Kit (Vector Laboratories, Burlingame, CA). The slides were counterstained with hematoxylin (Fisher Scientific Company, Kalamazoo, MI), dehydrated and mounted with Permount.

### Acridine orange assay

Dye was added to the culture and monitored on the Zeiss LSM710 microscope. *Helicobacter pylori* was added to the medium of the monolayers at a concentration of 50 μL of DMEM/F12 (1263–010, Gibco Life Technologies) containing 5–8 million bacteria. Histamine was added to the medium of 3D huFGOs, iPSC-derived HGO or 2D gastric epithelial monolayers at a concentration of 6.67 mM (Sigma Aldrich). Images were analyzed using the Zeiss LSM710 Microscope and background corrected 550-620/620-700 nm ratio values were converted to fold change corresponding to pH change using Prisim Graph Pad software.

### Western blots

Organoids and monolayers were harvested in cold DMEM/F12 and lysed in M-PER Mammalian Protein Extraction Reagent (Thermofisher) supplemented with protease inhibitors (Roche) according to the manufacturer’s protocol. Cell lysates were suspended in 40 μL of Laemmli Loading Buffer containing β-mercaptoethanol (BioRad). Samples containing 20 μg of protein were then loaded onto 1 4–20% Tris-Glycine Gradient Gels (Invitrogen) and run at 120 V for 1.5 hours before transferring the protein onto nitrocellulose membranes (Whatman Protran, 0.45 μM) at 105 V for 1.5 hours at 4°C. Membranes were blocked for 1 hour at 23°C using KPL Detector Block Solution (Kirkegaard & Perry Laboratories, Inc.). Next membranes were incubated overnight at 4°C with a 1:1000 dilution of anti-PD-L1 (Novus, NBP1-76769) a 1:1000 dilution of anti-Shh (Novus, AF464) or 1:2000 dilution of anti-GAPDH (Millipore, MAB374). The membranes were washed 3 times for 5 minutes each. Following this, the membranes were incubated with a 1:1000 dilution anti-mouse, 1:1000 dilution anti-goat or anti-rabbit Alexa Fluor 680 (Invitrogen). The blots were then imaged using a scanning densitometer along with analysis software (Odyssey Infrared Imaging Software System).

### Quantitative RT-PCR

Total RNA was isolated from tissue, glands, 3D organoids, HGOs and 2D organoid monolayers using TRIzol (Life Technologies) according to manufacturer’s protocol. A High Capacity cDNA Reverse Transcription Kit synthesized cDNA from 100 ng of RNA following protocol provided by Applied Biosystems. Real-time PCR assays were utilized for the following genes in HGOs and 2D organoid monolayers: GAPDH (Hs02786624_g1), PD-L1 (Hs01125296_m1), SHH (Hs00179843_m1), TFF2 (Hs00193719_m1), Clustrin (Hs00156548_m1), and HE4 (Hs00899484_m1). Cell lineage markers were determined in tissue, glands, hFGOs, 2D monolayers fundic and antral HGOs by RT-PCR for Mucin 5AC (Hs01365616_m1), Mucin 6 (Hs01674026_g1), H^+^/K^+^ ATPase ATP4B (Hs01026288_m1), Pepsinogen C (Hs00160052_m1) Pepsinogen A (Hs05416800_g1) and Mist 1 (Hs00703572_s1). PCR amplifications were done with a pre-validated 20X TaqMan Expression Assay primers and a 2X TaqMan Universal Master Mix (Applied Biosystems) and a cDNA template in a total volume of 20 μL. Amplifications were performed in duplicate wells in a StepOne Real-Time PCR System (Applied Biosystems). Fold change was calculated at (Ct-Ct high) = n target, 2ntarget/2nGAPDEH = fold change where Ct = threshold cycle.

### Flow cytometry

The media was removed from huFGO co-cultured with immune cells and infected or uninfected with *H*. *pylori*. The cultures were treated with accutase for 10 minutes at 37^o^ C. The organoids were then passed through a 27 1/8-gauge syringe in order to dissociate the organoids into single cells. CTLs were extracted using the EasySep Human CD8 Positive Selection Kit II (STEMCELL, 17853). Breifly, cells were incubated with 100 μL/mL of sample selection cocktail and incubated at room temperature for 3 minutes. 50 μL/mL of sample RapidSpheres were added to the sample and incubated at room temperature for 3 minutes. Samples were topped up to 5 mL with EasySep Buffer (STEMCELL, 20144) and incubated at room temperature for 3 minutes. Cells that did not bind to the magnetic beads were collected in a 50 mL conical. Samples were wash two more times with 5 mL of Easy Sep Buffer (STEMCELL, 20144). Cells that were adherent to magnetic beads were CTLs and were collected and centrifuged at 300 *g* for 5 minutes. Cells that did not bind to the magnetic beads were DCs and epithelial cells. These cells were also centrifuged at 300 *g* for 5 minutes. Fluoresence Assisted Cell Sorting was using to collect epithelial cells from the epithelial cell/DC mixture. This cell mixture was stained with EpCam (Biolegend) and CD11c (Biolegend). EpCam positive cells were collected during sorted and CD11c cells were disgarded. EpCam positive cells were suspended in 100 μL of a 1:1000 dilution of the zombie red cocktail (BioLegend). 1 μL of a 1:1000 dilution of the calcein violet cocktail (BioLegend) was added to this cell suspension. The cells were incubated in this cocktail for 20 minutes at room temperature. The cells were then washed with 1 mL of 5% BSA at 300 *g* for 5 minutes. The cells were then suspended in 100 μL of 5% BSA, treated with 1 μL of anti-PD-L1(BioLegend) and incubated for 15 minutes at room temperature. The cell suspension was then incubated at room temperature for 15 minutes with 100 μL of Reagent A (Thermofisher). The cells were then washed with 1 mL of 5% bovine serum albumin at 300 *g* for 5 minutes. CTLs that were extracted from culture were suspended in 100 μL of 5% BSA, treated with 1 μL of anti-CD8 (BioLegend) and anti-PD-1 (BioLegend) and incubated for 15 minutes at room temperature. The cells were then incubated at room temperature for 15 minutes with 100 μL of Reagent A (Thermofisher). The cells were then washed with 1 mL of 5% bovine serum albumin at 300 *g* for 5 minutes. The cells were suspended in 100 μL of Reagent B (Thermofisher) and 1 μL of anti-IL2 and 1 μL of anti-IFN-γ were added to the cells. This cocktail was incubated at room temperature for 20 minutes. The cells were washed with 1 mL of 5% bovine serum albumin at 300 *g* for 5 minutes. All cells were then suspended in 500 μL of 5% bovine serum albumin. Samples were run on the CANTO 3 and analyzed by FlowJo data analysis.

### Statistical analysis

The significance of the results was tested by one-way ANOVA, two-way ANOVA or student’s *t*-test using commercially available software (GraphPad Prism, GraphPad Software, San Diego, CA). A P value <0.05 was considered significant.
